# Physiological and Molecular Mechanisms of Differential Sensitivity of Palmer Amaranth (*Amaranthus palmeri*) to Mesotrione at Varying Growth Temperatures

**DOI:** 10.1371/journal.pone.0126731

**Published:** 2015-05-19

**Authors:** Amar S. Godar, Vijaya K. Varanasi, Sridevi Nakka, P. V. Vara Prasad, Curtis R. Thompson, J. Mithila

**Affiliations:** Department of Agronomy, Kansas State University, Manhattan, Kansas, United States of America; Ghent University, BELGIUM

## Abstract

Herbicide efficacy is known to be influenced by temperature, however, underlying mechanism(s) are poorly understood. A marked alteration in mesotrione [a 4-hydroxyphenylpyruvate dioxygenase (HPPD) inhibitor] efficacy on Palmer amaranth (*Amaranthus palmeri* S. Watson) was observed when grown under low- (LT, 25/15°C, day/night temperatures) and high (HT, 40/30°C) temperature compared to optimum (OT, 32.5/22.5°C) temperature. Based on plant height, injury, and mortality, Palmer amaranth was more sensitive to mesotrione at LT and less sensitive at HT compared to OT (ED_50_ for mortality; 18.5, 52.3, and 63.7 g ai ha^-1^, respectively). Similar responses were observed for leaf chlorophyll index and photochemical efficiency of PSII (*F_v_/F_m_*). Furthermore, mesotrione translocation and metabolism, and *HPPD* expression data strongly supported such variation. Relatively more mesotrione was translocated to meristematic regions at LT or OT than at HT. Based on T_50_ values (time required to metabolize 50% of the ^14^C mesotrione), plants at HT metabolized mesotrione faster than those at LT or OT (T_50_; 13, 21, and 16.5 h, respectively). The relative *HPPD*:*CPS* (carbamoyl phosphate synthetase) or *HPPD*:*β*-tubulin expression in mesotrione-treated plants increased over time in all temperature regimes; however, at 48 HAT, the *HPPD*:*β*-tubulin expression was exceedingly higher at HT compared to LT or OT (18.4-, 3.1-, and 3.5-fold relative to untreated plants, respectively). These findings together with an integrated understanding of other interacting key environmental factors will have important implications for a predictable approach for effective weed management.

## Introduction

Palmer amaranth (*Amaranthus palmeri* S. Watson) is a C_4_, summer annual, dioecious broad-leaved plant and is the most economically damaging and troublesome weed in major crops grown in the USA [1–5]. Increasing occurrence of herbicide resistance in several biotypes of this weed (dinitroanilines [6]; acetolactate synthase (ALS)-inhibitors [7]; glyphosate [8]; triazines and 4-hydroxyphenylpyruvate dioxygenase (HPPD)-inhibitors [9]) pose serious crop production challenges in the USA. In addition, Palmer amaranth populations resistant to multiple herbicide modes of action have been reported [9,10].

More efficient use of herbicides is increasingly important because of the stagnation in the discovery of new herbicide modes of action in the past decade and an alarming rate of increase in evolution of herbicide-resistant weeds globally, leaving fewer weed control options. Thus, future weed management strategies will deploy different herbicides with different modes of action in sequences, mixtures, or rotations [11]. In this scenario of shifting weed management strategies, HPPD-inhibitors (e.g. mesotrione) are viewed as vital tools for managing weeds in various situations. [12–14]. In addition, herbicidal potential of HPPD-inhibiting herbicides has raised interest in developing resistant transgenic crops [15]. For several reasons as stated above, Palmer amaranth is one of the most troublesome weeds of the US agriculture [5], therefore, a better understanding of potential environmental factors, specifically growth temperature, which can influence the efficacy of mesotrione on its control is crucial.

Mesotrione (2-[4-(methylsulfonyl)-2-nitrobenzoyl]-1,3-cyclohexanedione) is a triketone herbicide [16] and inhibits the HPPD enzyme, a critical component of the tocopherol biosynthesis pathway that converts tyrosine to plastoquinone (PQ) and α-tocopherol [17]. PQ is an essential component of carotenoid biosynthesis [18] and the limited or no availability of PQ affects the downstream synthesis of carotenoids, which are essential for two critical roles during photosynthesis; light harvesting and protection against photooxidative damage [19]. Tocopherol has antioxidant functions; quenching and scavenging of reactive oxygen species (ROS) such as ^1^O_2_ and OH^-^ radicals [19–21]. Thus, mesotrione treatment leads to burst of ROS production in sensitive species.

A better understanding of how weed species respond to herbicides under changing environmental conditions aids in development of viable weed management strategies. Several studies have shown that growth temperature greatly affects herbicide efficacy [22–28]. While, studies have reported greater efficacy of certain herbicides e.g. glyphosate, metriflufen, acifluorfen on bermudagrass (*Cynodon dactylon* L. Pers.), johnsongrass (*Sorghum halepense* L. Pers.) and soybeans (*Glycine max* L. Merr.) under higher temperatures [29–33], few recent studies found opposite results with herbicides such as mesotrione [26,34]. Such findings indicate that the effect of temperature on herbicide efficacy largely depends on the herbicide chemistry and weed species in question. Moreover, underlying mechanism(s) of such alerted efficacy of herbicide under varying temperatures is poorly understood and needs investigation for better management of weeds.

In this study we demonstrate the physiological and molecular basis of differential mesotrione efficacy on Palmer amaranth under varying growth temperatures. Mesotrione dose-response, absorption, translocation and metabolism, and *HPPD* gene expression were studied in a mesotrione-susceptible Palmer amaranth population from Mississippi, USA grown at three [low, optimum (normal temperature during summer months in Kansas, USA and neighboring states), and high] temperature regimes.

## Materials and Methods

### Plant material and growth conditions

Palmer amaranth population from Mississippi, USA susceptible to mesotrione was used to investigate the effect of temperature on efficacy of POST-applied mesotrione. Seeds of Palmer amaranth were germinated in small trays (25 x 15 x 2.5-cm) with commercial potting mixture (Miracle Gro, Marysville, OH, USA) and individual seedlings (2 to 3 cm tall, two-leaf stage) were transplanted into plastic pots (6 x 6 x 6.5-cm) in a greenhouse maintained at 25/20°C, day/night, 60% relative humidity, and 15/9 h day/night photoperiod, supplemented with 120 μmol m^-2^ s^-1^ illumination provided with sodium vapor lamps. After 6 to 7 days of transplanting, healthy uniform sized plants (~5 cm tall, four-leaf stage) were transferred to growth chambers that were maintained at different temperature regimes; low (LT; 25/15°C, day/night), optimum (OT; 32.5/22.5°C, day/night) and elevated (HT; 40/30°C, day/night). Light in the growth chamber was provided by incandescent and fluorescent bulbs delivering 550 μmol m^-2^ s^-1^ photon flux (15/9 h day/night) at plant canopy level. All the growth chambers were set to maintain 60% relative humidity throughout the experiment. Plants were watered regularly as needed and were fertilized one week after transplanting.

### Mesotrione dose-response study

Treatments were replicated four times in each experiment and the complete experiment was conducted two times (repeated over time) except for plant biomass.

#### Mesotrione treatment

Palmer amaranth plants were treated with different doses of mesotrione when the plants were 10 to 12 cm tall (8 to 10 leaf stage). Mesotrione (Callisto, Syngenta) was applied at 0, 3.28, 6.563, 13.125, 26.25, 52.5, 105, and 210 g ai ha^-1^. All treatments included crop oil concentrate (COC, Agridex, USA) and ammonium sulphate (AMS, Liquid N-PAK; Agriliance, USA) at 1% v/v and 0.85% w/v, respectively. Treatments were applied with a bench-type sprayer (Research Track Sprayer, De Vries Manufacturing, RR 1 Box 184, Hollandale, MN, USA) equipped with a flat-fan nozzle tip (80015LP TeeJet tip, Spraying Systems Co., Wheaton, IL, USA) delivering 168 L ha^-1^ at 222 kPa in a single pass at 4.8 km h^-1^. Temperature, relative humidity, and light intensity at the time of mesotrione treatment were 25°C, 60%, and 10 μmol m^-2^ s^-1^, respectively. Following treatment, plants were returned to corresponding growth chambers (within 30 min after treatment).

#### Visual injury, plant mortality, and biomass measurement

Injury ratings were based on composite visual estimations of growth inhibition, bleaching, necrosis, and plant vigor with the use of a scale of 0 (no effect) to 100 (plant death). For plant mortality count, plants injured 0 to 97% were considered alive and those injured > 97% were considered dead. Plant height, visual injury, and survival count were taken at 1, 2, and 3 weeks after treatment (WAT). Plants were clipped off at the base (~1 cm above soil surface), immediately weighed (aboveground fresh biomass) 4 WAT. Harvested plants were collected in separate paper sacks and were weighed (dry biomass) following oven drying at 60°C for 72 h.

#### Chlorophyll index and *F*_*v*_ */F*_*m*_ measurement

Chlorophyll index and *F*_*v*_ */F*_*m*_ were measured on the middle of the upper surface of the fourth leaf from the top of the plant avoiding the midrib. Chlorophyll index was measured using a chlorophyll meter (SPAD 502 Plus Chlorophyll Meter, Spectrum Technologies, Inc., Aurora, IL, USA). A chlorophyll fluorometer (OS-30p, Opti Sciences Inc., Hudson, NH, USA) was used to measure the photochemical efficiency of PSII (*F*_*v*_ */F*_*m*_) under light-adapted condition. Both measurements were taken at 1, 2, and 3 WAT.

### Absorption and translocation of [^14^C] mesotrione

Greenhouse grown seedlings (as described above) of Palmer amaranth were moved to growth chambers maintained at LT, OT and HT 4 to 5 days before herbicide treatment to allow plants to acclimate. Ten to 12 cm tall (8 to10 leaf stage) plants were treated with four 2.5-μL droplets of [phenyl-U-^14^C]-mesotrione (3.3 kBq with specific activity of 781 MBq g^-1^) on the upper surface of the fourth youngest leaf. Unlabeled mesotrione was added to the radioactive solution to obtain 105 g ai mesotrione in a carrier volume of 187 L. Crop oil concentrate (COC, Agridex, USA) and ammonium sulphate (AMS, Liquid N-PAK; Agriliance, USA) were added at 1% v/v and 0.85% w/v respectively as adjuvants to enhance droplet-to-leaf surface contact. After 30 min, plants were returned to the growth chambers. Plants were harvested at 8, 24, 48 and 72 h after treatment (HAT) and separated into treated leaf (TL), tissue above the treated leaf (ATL), or below the treated leaf (BTL). Treated leaves were washed in a 20-mL scintillation vial with 5 mL wash solution (10% methanol and 0.05% polysorbate 20) for 1 min. Radioactivity in the leaf rinsate was measured by using liquid scintillation spectrometry (LSS). Plant sections were dried at 60°C for 48 h and total radioactivity absorbed in each plant part was quantified by combusting with a biological oxidizer (OX-501, RJ Harvey Instrument, New York, NY, USA) and liquid scintillation spectrometry (Tricarb 2100 TR Liquid Scintillation Analyzer; Packard Instrument Co., Meriden, CT, USA). Herbicide absorption was calculated as; % absorption = (total radioactivity applied—radioactivity recovered in wash solution) x 100 / total radioactivity applied. Herbicide translocation was determined as; % translocation = 100 –% radioactivity recovered in treated leaf, where % radioactivity recovered in treated leaf = radioactivity recovered in treated leaf x 100 / radioactivity absorbed. Treatments were replicated four to five times and the experiment was repeated.

### Metabolism of mesotrione in whole plants

Eight 2.5-μL droplets containing a total of 7.2 kBq of [^14^C] mesotrione were applied to the upper surface of the fourth and fifth youngest leaves on each plant (10 to 12 cm tall, 8 to 10 leaf stage). Unlabeled mesotrione was mixed with [^14^C] mesotrione to reach the desired concentration of 105 g ai in 187 L carrier volume as described above for the absorption and translocation study. [^14^C] mesotrione and its metabolites were extracted with modification to methods described by Ma et al. [35]. At 8, 24, 48 and 72 HAT, the treated leaves were harvested and washed as described above for the absorption and translocation study. Whole plant tissue and the washed treated leaves were then frozen in liquid nitrogen and homogenized with a mortar and pestle. [^14^C] mesotrione and its metabolites were extracted with 15 mL of 90% acetone at 4°C for 16 h. Samples were centrifuged at 6500 rpm (5000 *g*) for 10 min. Supernatant was concentrated at 45°C for 2 to 3 h depending on the rate of evaporation until a final volume of 500 μL was reached with a rotary evaporator (Centrivap, Labconco, Kansas City, MO, USA). About 500 μL of extract was transferred to a 1.5-mL microcentrifuge tube and centrifuged at high speed (13000 rpm/10000 *g*) for 10 min. The total radioactivity in each sample was measured by LSS prior to HPLC analysis and samples were normalized to 120 dpm μL^-1^ (amount of [^14^C] compounds) by diluting the samples with 50:50 (v/v) acetonitrile:water.

Total extractable radioactivity in 50 μL of the samples was resolved into parent mesotrione and its polar metabolites by reverse-phase High-performance Liquid Chromatography (HPLC) (System Gold, Beckman Coulter, Pasadena, CA, USA) as described below. Reverse-phase HPLC was performed with a Zorbax SB-C18 column (4.6 x 250 mm, 5-μm particle size; Agilent Technologies, Santa Clara, CA, USA) at a flow rate of 1 mL min^-1^ with eluent A (water with 0.1% trifluoroacetic acid, TFA) and eluent B (acetonitrile with 0.1% TFA). The elution profile was as follows: 0 to 2 min, 0 to 20% (of eluent B) linear gradient; 2 to 10 min, 20 to 40% linear gradient; 12 to 17 min, 40 to 70% linear gradient; 17 to 19 min, 70 to 90% linear gradient (19 min total) followed by; 19 to 22 min, 90 to 50% linear gradient; 22 to 25 min, 20% isocratic hold to re-equilibrate the column for the next sample injection (25 min total). The retention time of the parent compound, [^14^C] mesotrione, was determined by injecting 50- μL of 150 dpm μL^-1^ [^14^C] mesotrione diluted in 1:1 acetonitrile:water. The parent compound displayed a retention time of 18.3 min. The parent compound and other radiolabeled metabolites were detected with a radioflow detector (EG & G Berthold, LB 509, Bad Wildbad, Germany) and Ultima-Flo M cocktail (PerkinElmer, Waltham, MA, USA). The amount of parent herbicide, [^14^C] mesotrione, remaining was quantified as a percentage of total extractable radioactivity, based on peak area determined. Treatments were replicated three times and the experiment for 48 and 72 h harvest times was repeated.

### *HPPD* gene expression

Fresh leaf tissue after mesotrione treatment at 105 g ai ha^-1^ as previously described was collected at different time points 0, 4, 8, 24, and 48 HAT from Palmer amaranth plants growing at LT, OT, and HT and flash frozen in liquid nitrogen (-196°C). The collected tissue was stored at -80°C for RNA isolation. The frozen tissue was homogenized in liquid nitrogen using a pre-chilled mortar and pestle to prevent thawing. The powdered tissue was transferred to a 1.5 mL microcentrifuge tube and total RNA was isolated using RNeasy Plant Mini Kit (Qiagen Inc., Valencia, CA, USA). RNA was treated with DNase 1 enzyme (Thermo Scientific, Waltham, MA, USA) to remove genomic DNA contamination. The isolated RNA was stored at -80°C. The quantity and quality of total RNA was determined using a spectrophotometer (NanoDrop 1000, Thermo Scientific) and agarose gel (1%) electrophoresis.

cDNA was synthesized from 1 μg of total RNA using RevertAid First Strand cDNA Synthesis Kit (Thermo Scientific). The synthesized DNA was diluted in 1:5 ratio and used in quantitative PCR (qPCR) reaction. The qPCR reaction mix consisted of 8 μL of SYBR Green mastermix (BioRad Inc., Hercules, CA, USA), 2 μL each of forward and reverse primers (5 μmoles), and 2 μL of the diluted cDNA to make the total reaction volume of 14 μL. *HPPD* gene expression was normalized using either *CPS* (carbamoyl phosphate synthetase) or *β*-tubulin as a reference gene. PCR conditions were 50°C for 2 min, 95°C for 10 min, and 40 cycles of 95°C for 30 s and 60°C for 1 min [35]. A meltcurve profile was included following the thermal cycling protocol to determine the specificity (no primer dimers, no genomic DNA contamination, and no non-specific product) of the qPCR reaction. Primer sequences used were: HPPDF 5’-CTGTCGAAGTAGAAGACGCAG-3’ and HPPDR 5’-TACATACCGAAGCACAACATCC-3’ [35]; CPSF 5’-ATTGATGCTGCCGAGGATAG-3’ and CPSR 5’- GATGCCTCCCTTAGGTTGTTC-3’ [35]; *β*-tubulinF 5'-ATGTGGGATGCCAAGAACATGATGTG-3' and *β*-tubulinR 5'-TCCACTCCACAAAGTAGGAAGAGTTCT-3'. The *β*-tubulin gene sequences of *Sesamum indicum* (LOC105162689), *Populus euphratica* (LOC105136926), *Elaeis guineensis* (LOC105045457), and *Tarenaya hassleriana* (LOC104800466) obtained from GenBank were used to design conserved gene specific primers. The *HPPD*, *CPS*, and *β*-tubulin primers showed efficiency within 100±10% (tested with four 5-fold serial dilutions) and both the reference genes were stably expressed (<1 fold magnitude of differences in CT values) under the specific experimental conditions (0, 4, 8 and 24 HAT for *CPS* and 0 and 48 HAT for *β*-tubulin) used in this study. The qPCR was performed using CFX96 Touch Real-Time PCR Detection System (BioRad Inc.). The *HPPD*:*CPS* or *HPPD*:*β*-tubulin expression was determined using the 2^ΔCT^ method, where CT is threshold cycle and ΔCT is CT_Reference gene (*CPS or β-t*ubulin)—_CT_Target gene (*HPPD*)_. Gene expression was studied on two to three biological and three technical replicates.

### Statistical analysis

In the whole-plant dose response study, the treatments were arranged in a complete factorial combination of three levels of growth temperatures (LT, OT, and HT) and eight levels of mesotrione rates (see materials and methods section). Plant height, visual injury, chlorophyll index, and *F*_*v*_ */F*_*m*_ data were subjected to non-linear regression analysis in order to estimate ED_50_ values as described by [36]. Specifically, three parameter log-logistic model for plant height, injury, and mortality, and Weibull model for chlorophyll index and *F*_*v*_ */F*_*m*_ data were used (lack of fit test P > 0.05). There was no interaction between the experimental runs and the treatments, hence, the data of the two dose-response studies (each with 4 replications) were pooled prior to analysis. The magnitude of differences in responses of plants grown under different temperature regimes was measured as the sensitivity index (SI) (OT/HT or OT/LT ratio) of estimated ED_50_, ED_80_, or ED_85_ values using the SI function in the *drc* package in R v.3.1.0 [36].

For experiments involving mesotrione absorption and translocation, metabolism, and *HPPD* expression, the treatments were arranged in complete factorial combination of three levels of growth temperatures (LT, OT and HT), as main factors, and 4 to 5 levels of measurement time points, as simple factors. There was no interaction between the experimental runs and the treatments, hence, the data from each independent experiment were combined before subjecting to statistical analysis. Data for these experiments were analyzed by two-way ANOVA (P < 0.05). Data for all the experiments were significant for the main effects (growth temperatures), hence, post-hoc Tukey-HSD pairwise comparisons were used to test which simple effects were significantly different (P < 0.05).

## Results

### Plant height, visual injury, and plant mortality

**Fig 1:**
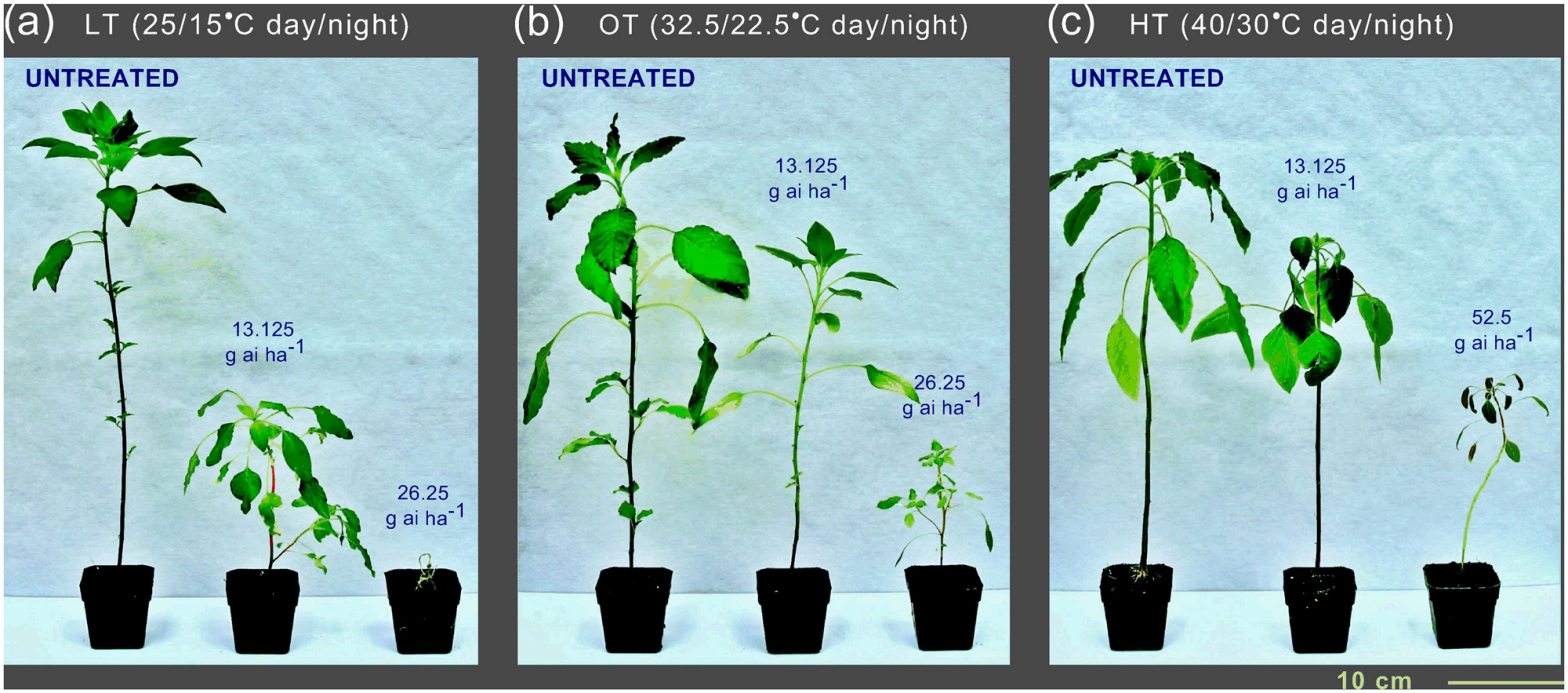
Photographs of mesotrione-treated Palmer amaranth plants grown under (a) LT (25/15°C, day/night), (b) OT (32.5/22.5°C, day/night), and (c) HT (40/30°C, day/night) conditions (15/9 h day/night). Plant to plant variability was observed within the growth temperature and mesotrione rate. These are the representative plants for each dose and temperature. The photographs were taken 4 weeks after treatment and all photographs were taken under the same magnification.

**Fig 2:**
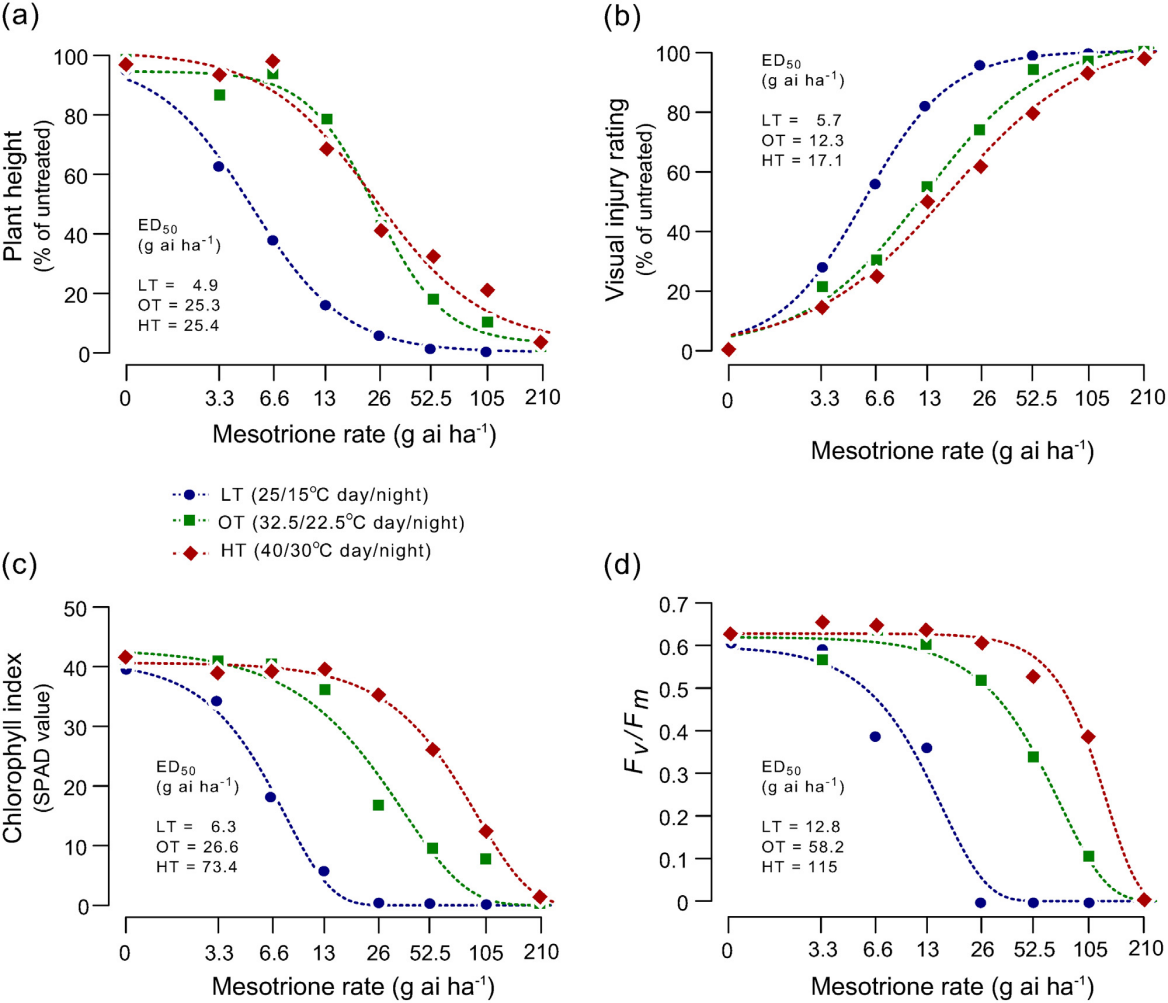
Whole-plant mesotrione dose-response of Palmer amaranth at different temperatures (low temperature, LT, 25/15°C; optimum temperature, OT, 32.5/22.5°C; and high temperature, HT, 40/30°C; 15/9 h day/night) as measured by (a) plant height 3 weeks after treatment (WAT), (b) visual injury 3 WAT, (c) leaf chlorophyll index 2 WAT, and (d) photochemical efficiency of PSII (*F*_*v*_ */F*_*m*_) 2 WAT. Palmer amaranth plants (10 to12 cm tall, 8 to 10 leaf stage) were treated with 0, 3.28, 6.563, 13.125, 26.25, 52.5, 105, and 210 g ai ha^-1^ mesotrione with 1% v/v crop oil concentrate (COC) and 0.85% w/v ammonium sulphate (AMS). Curves for height and visual injury, and chlorophyll index and *F*_*v*_ */F*_*m*_ data were fitted using three parameter log-logistic and Weibull model, respectively, as described by Knezevic et al. (2007).

Four weeks after treatment (WAT), Palmer amaranth plants showed decreasing sensitivity to mesotrione as temperature increased from LT to HT (Fig 1). With an increase in growth temperature from LT to HT, a shift towards less sensitivity of Palmer amaranth to mesotrione was evident as indicated by plant height (Fig 2a) and visual injury (Fig 2b) 3 WAT and mortality 4 WAT (S1 Fig). Based on plant height, visual injury, and mortality response, Palmer amaranth plants were two- to five-times more sensitive to mesotrione when grown under LT compared to OT condition (S1, S2 and S3 Tables). Mesotrione rates that caused 50% height reduction (ED_50_) were 4.9, 25.3, and 25.4 g ai ha^-1^ under LT, OT, and HT, respectively (S1 Table). ED_50_ for injury were two- and one and half-times less for OT and HT, respectively (S2 Table). Plant survival results were in accordance with the visual injury results (S1 Fig; S3 Table). Although ED_50_ values for plant height were similar for OT and HT conditions, the ED_85_ values for HT was higher compared to the ED_85_ values under OT (S1 Table). Similar results were also observed for plant survival at 4 WAT (S1 Fig; S3 Table).

### Leaf chlorophyll index and photochemical efficiency of PSII

Application of mesotrione causes characteristic bleaching [16,37] and reduces photosynthetic efficiency of plants [38]. The chlorophyll index in plants grown under LT sharply decreased with the lowest doses of mesotrione reaching zero at 26.25 g ai ha^-1^ 2 WAT (Fig 2c), however, mesotrione rates ≤ 6.56 and ≤ 13.125 g ai ha^-1^ had no effect on chlorophyll index at OT and HT, respectively. The ED_50_ values (mesotrione rates that caused 50% chlorophyll reduction) were 6.3, 26.6 and 73.4 g ai ha^-1^ at LT, OT, and HT, respectively (S4 Table). At OT and HT, plants treated with the low mesotrione rates also showed some level of bleaching at 1 WAT, however, they recovered to normal within 2 WAT (Fig 2c).

The *F*_*v*_ */F*_*m*_ values of the plants grown under LT also declined exponentially with increasing mesotrione rate reaching zero at 26.25 g ai ha^-1^ 2 WAT (Fig 2d). In contrast, mesotrione rates ≤ 26.25 g ai ha^-1^ had no effect on *F*_*v*_ */F*_*m*_ values at HT. Plants at OT had an intermediate response of LT and HT. Mesotrione rates that caused 50% reduction in *F*_*v*_ */F*_*m*_ (ED_50_) were 12.8, 58.2 and 115 g ai ha^-1^ at LT, OT, and HT, respectively (S5 Table).

### Mesotrione uptake and translocation

**Fig 3:**
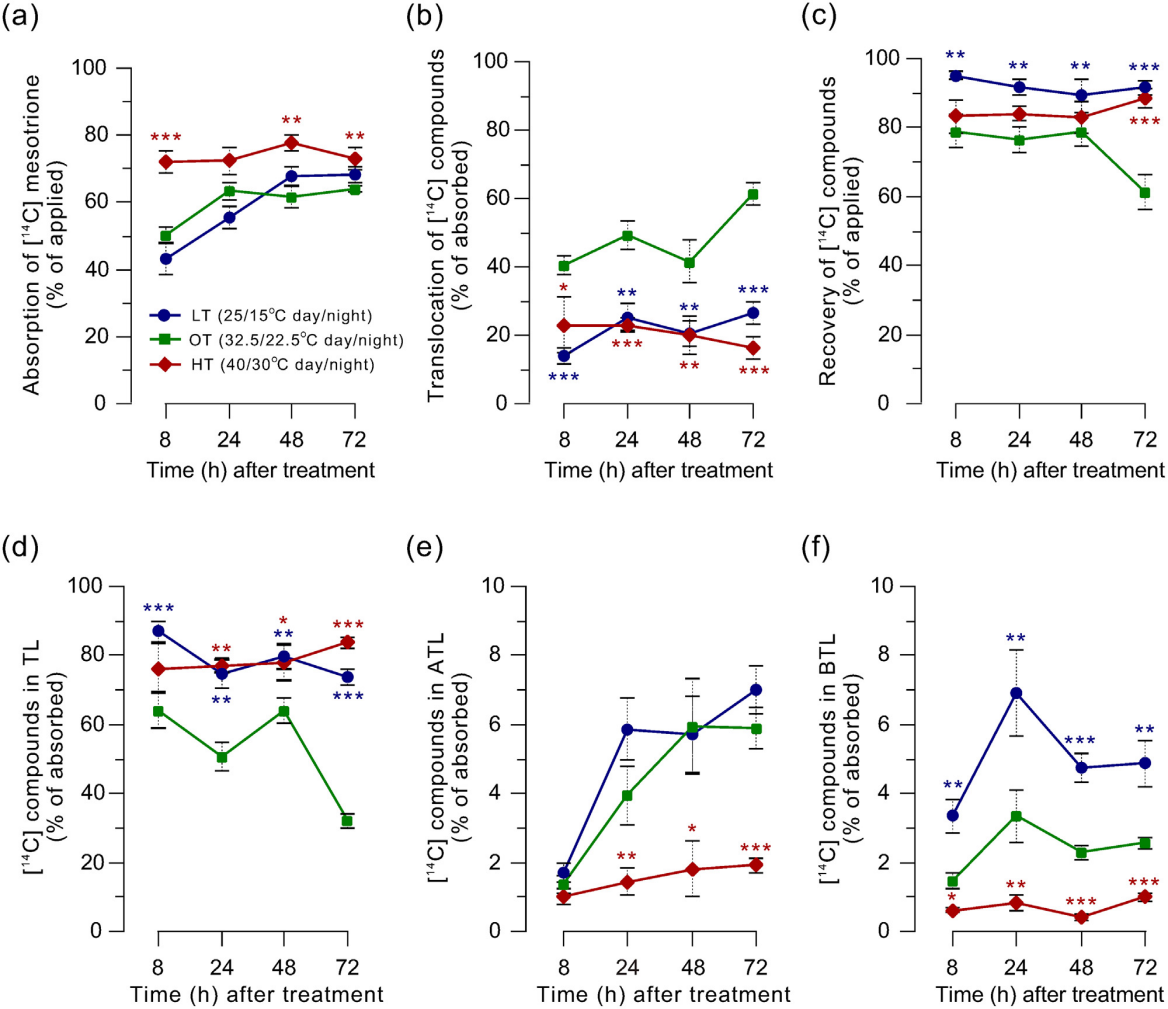
[^14^C] mesotrione absorption (a), translocation (b), total recovery (c), and translocation to treated-leaf (d), above treated-leaf (e) and below treated-leaf (f) at three different temperatures (low temperature, LT, 25/15°C; optimum temperature, OT, 32.5/22.5°C; and high temperature, HT, 40/30°C; 15/9 h day/night). The upper surface of fourth youngest leaf of Palmer amaranth plants (10 to 12 cm tall, 8 to 10 leaf stage) were treated with 4- x 2.5-μL droplets (1.6548 mM mesotrione, 0.85% w/v AMS, and 1% COC) containing 3.3 kBq of [^14^C] mesotrione. Significant differences (within harvest time) between the OT and LT (blue asterisks) or HT (red asterisks) plants are indicated with asterisks (*, P ≤ 0.05; **, P < 0.01). Error bars represent SE.

In susceptible plant species, mesotrione is rapidly absorbed by plants and translocates both acropetally and basipetally [16]. In Palmer amaranth, growth temperature influenced both absorption and translocation of [^14^C] mesotrione (Fig 3). More than 70% of mesotrione was absorbed (% of total applied) within the initial 8 HAT in plants grown under HT, whereas, only 43 and 50% mesotrione was absorbed at LT and OT, respectively (Fig 3a). No further mesotrione absorption occurred at HT, however, absorption continued to increase slowly at LT and OT reaching 68 and 64%, respectively at 72 HAT. Greater absorption of mesotrione did not lead to increased sensitivity of Palmer amaranth grown under HT, thus, the greater absorption is likely due to higher rates of metabolism of absorbed mesotrione within underlying leaf tissues resulting in greater driving force for more mesotrione absorption [39,40]. Translocation of [^14^C] mesotrione and/or its metabolites (% of absorbed) was highest under OT which was 40% 8 HAT and increased to 61% by 72 HAT (Fig 3b). In general, about two-fold less absorbed [^14^C] mesotrione and/or its metabolites were translocated from the treated-leaf to other parts of the plants grown under LT and HT compared to OT. Overall, total radioactivity recovered from the aboveground part (% of applied) was highest in plants grown under LT (> 90%), intermediate under HT (83 to 89%) and lowest under OT (< 80%) (Fig 3c). Fairly similar levels of radioactivity were recovered from plants grown under LT and HT across the time points, however, the lowest recovery always occurred in plants grown under OT, with a distinctive sharp decline from 79% at 48 HAT to 61% at 72 HAT. These results indicate more translocation of radioactivity to below ground part in plants grown at OT compared to those at LT and HT.

In plants grown under HT, the majority of absorbed radioactivity (77 to 83%) remained in the treated-leaf, with < 2% translocated to parts above treated-leaf (Fig 3d and 3e). A similar amount of radioactivity was recovered in the treated-leaf portion of plants grown under LT, however, greater than three-fold more radioactivity was translocated upward by 72 HAT compared to plants grown under HT. Only 32% radioactivity was recovered in the treated-leaf of plants at OT 72 HAT, and the upward translocation was similar to those at LT. On average, about 5, 2.5, and 1% of the absorbed radioactivity was recovered from plant parts below the treated-leaf at LT, OT, and HT, respectively (Fig 3f).

### Mesotrione metabolism in whole plants

**Fig 4:**
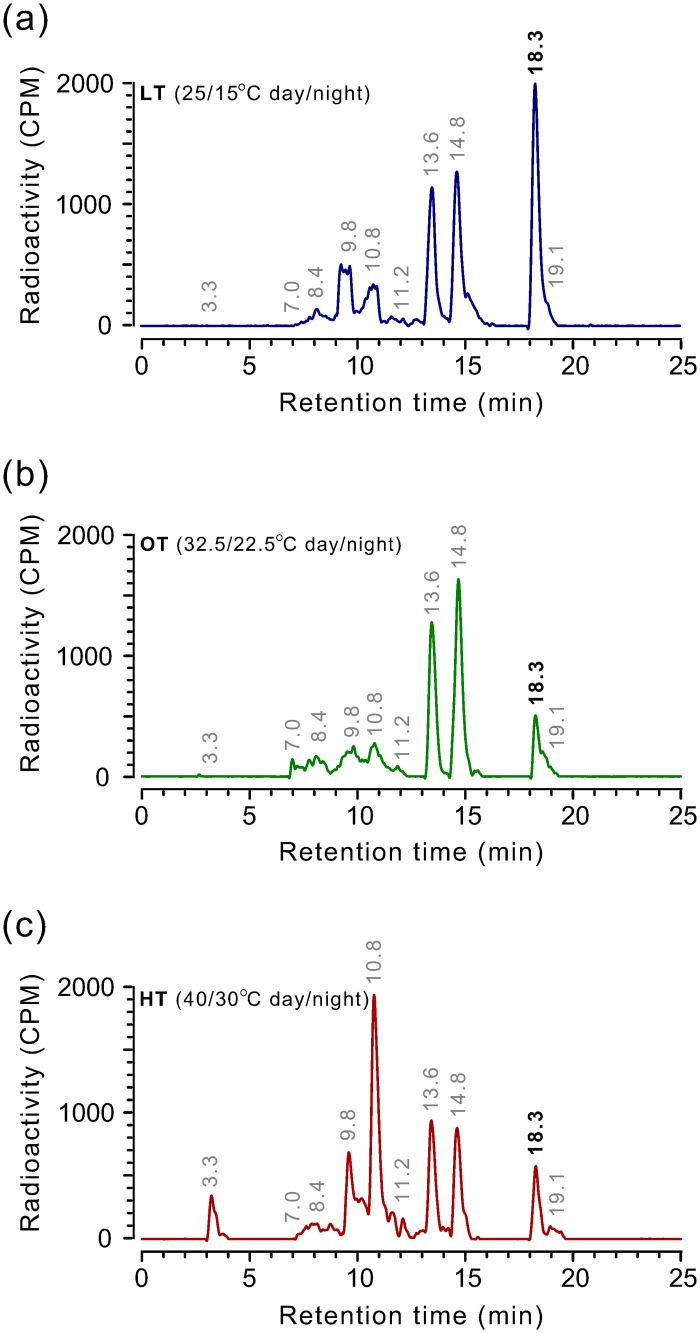
Representative reverse-phase HPLC chromatograms of mesotrione metabolism in Palmer amaranth plants grown under (a) LT (25/15°C day/night), (b) OT (32.5/22.5°C day/night, and (c) HT (40/30°C day/night) conditions (15/9 h day/night) at 48 h after treatment. Peak retention time around 18.3 min represents the parent mesotrione compound. Palmer amaranth plants (10 to 12 cm tall) were treated with 8- x 2.5-μL droplets (1.6548 mM mesotrione, 0.85% w/v AMS, and 1% COC) containing 7.2 kBq of [^14^C] mesotrione on the upper surface of fourth and fifth youngest leaves. Numbers above the peaks represent retention time (min).

Rate of mesotrione metabolism differs among plant species and the ability of some crops (e.g. *Zea mays* L., maize) to rapidly metabolize mesotrione offers acceptable selectivity against weeds [17]. Parent mesotrione (18.3 min retention time) was clearly resolved from several polar metabolites by reverse phase HPLC (Fig 4). The peak area of parent mesotrione in LT samples was higher than those from OT or HT at 48 HAT (Fig 4a, 4b and 4c). In OT and HT samples, the peak area of the parent mesotrione was comparable, but a peak at 10.8 min retention time was more prominent in HT than in LT and OT samples. This peak is possibly a hydroxylated form of mesotrione found in maize and waterhemp (4-hydroxy-mesotrione) [35,41] and its accumulation is likely due to enhanced cytochrome P450 (CYP) monooxygenases (P450) activity. P450s are membrane bound enzymes and are well known for playing a major role in metabolism of xenobiotics including mesotrione.

**Fig 5:**
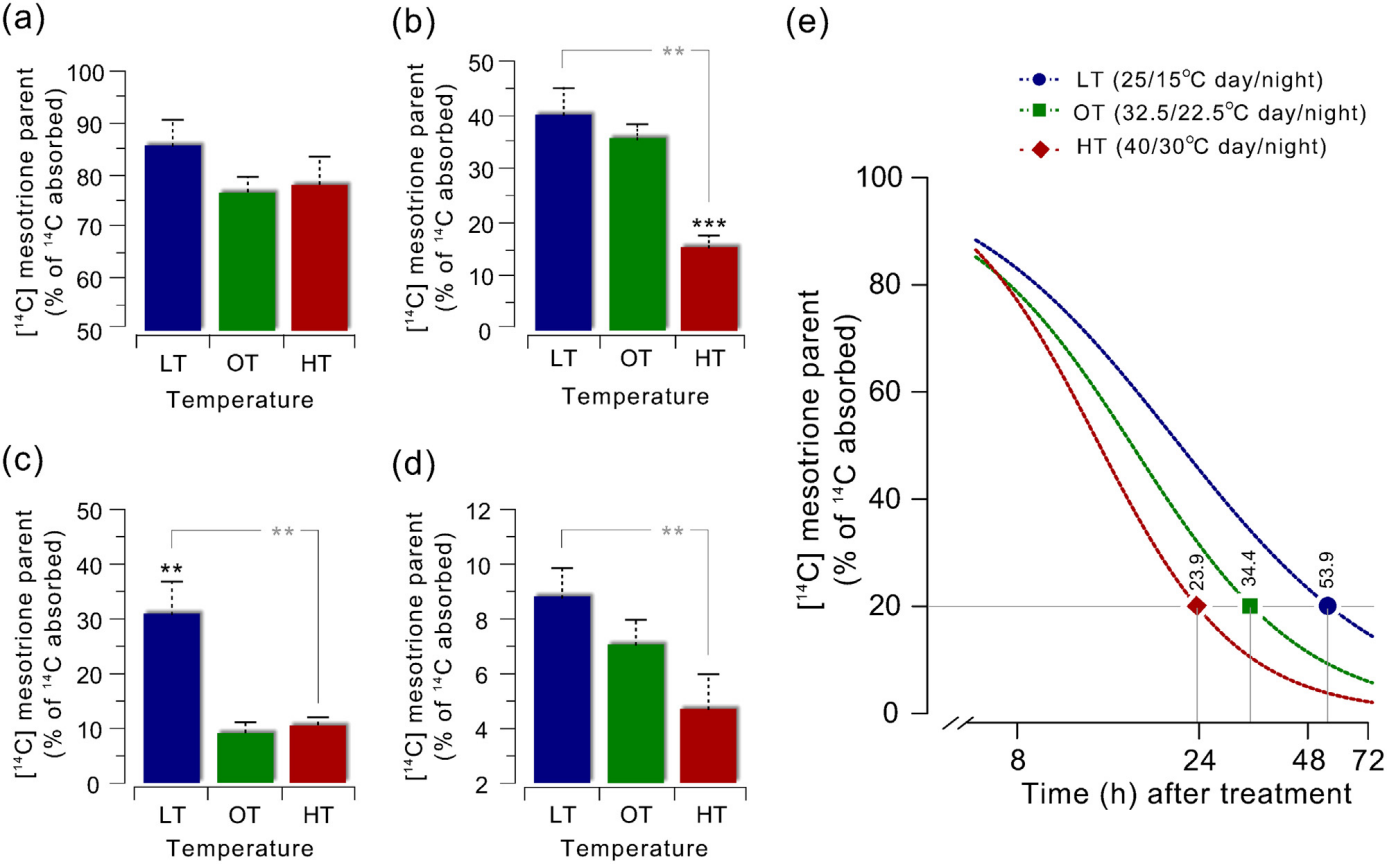
Parent [^14^C] mesotrione remaining in Palmer amaranth (whole aboveground part) plants under three different temperatures (low temperature, LT, 25/15°C; OT, optimum temperature, OT, 32.5/22.5°C; and high temperature, HT, 40/30°C; 15/9 h day/night). (a) 8, (b) 24, (c) 48, (d) 72 h after treatment as determined by reverse-phase HPLC, and (e) time course of mesotrione metabolism. All Palmer amaranth plants (10 to 12 cm tall, 8 to 10 leaf stage) were treated with 8- x 2.5-μL droplets (1.6548 mM mesotrione, 0.85% w/v AMS, and 1% COC) containing 7.2 kBq of [^14^C] mesotrione on the upper surface of fourth and fifth youngest leaves. Data represent means of three to five biological samples. Significant differences in mean values between OT and HT or LT are indicated with dark color asterisks, and between HT and LT with light color asterisks (*, P ≤ 0.05; **, P < 0.005). Error bars represent SE. Curves for [^14^C] parent compound (mesotrione) data were fitted using a three parameter log-logistic model.

Metabolism of mesotrione in Palmer amaranth did not differ among growth temperature conditions 8 HAT, and only 15 to 25% of the parent compound was metabolized (Fig 5a). At 24 HAT, the parent compound in HT samples was less (15.4%) compared to the parent compound found in OT (35.1%, P = 0.003) and LT samples (40%, P = 0.014) (Fig 5b). Plants grown under LT did not further metabolize mesotrione at 24 h through 48 h (30.5%, P = 0.2) (Fig 5c). However, approximately 3-times less parent compound remained in plants grown under OT (8.8%, P = 0.002) and HT (10%, P = 0.001) 48 HAT compared to the parent compound in LT. By 72 HAT, plants grown under all temperatures metabolized more than 90% of the parent compound (Fig 5d). The mean parent compound remaining was least in plants grown under HT (4%) which was similar to OT plants (7.56%, P = 0.154), but two-times less compared to LT (8.9%, P = 0.023). These results indicate that mesotrione metabolism in Palmer amaranth was enhanced at HT compared to OT, and it was reduced at LT.

To estimate the rate of [^14^C] mesotrione metabolism at LT, OT and HT growth conditions, we analyzed the metabolism data using a log-logistic regression (Fig 5e). The T_50_ (the estimated time point for 50% [^14^C] mesotrione metabolism) values were 21 and 13 h for LT and HT, respectively, and were not different from OT (16.5 h) (P = 0.14, LT; P = 0.2, HT) (S6 Table). The estimated T_50_ for mesotrione metabolism in this Palmer amaranth population when grown under HT is similar to that of mesotrione-resistant waterhemp (*A*. *tuberculatus*) in which enhanced metabolism of mesotrione was attributed for the resistance [35]. There were differences in time taken for 80% metabolism (T_80_) among the growth temperatures. The T_80_ value was lowest for HT plants (23.9 h), followed by OT (34.4 h) and LT (53.9 h) (S6 Table). The significantly longer T_80_ values for LT and shorter values for HT plants relative to OT plants are in conformity with the whole-plant dose-response results (Fig 2 and S1, S2, S3, S4 and S5 Tables).

### Target-Site Gene (*HPPD*) Expression

No experiments have been conducted to study the expression of *HPPD* gene in mesotrione-treated plants. In arabidopsis, *HPPD* was up-regulated under a high-light stress condition [42]. When exposed to excessive light, plants inactivate photosynthetic functions and produce ROS. Even in normal light conditions, plants treated with HPPD-inhibitors experience light-stress as HPPD inhibition results in decreased carotenoid and tocopherol content in plants. We hypothesized (1) *HPPD* is up-regulated in plants when treated with mesotrione and (2) *HPPD* expression is greater in the mesotrione treated-plants grown at high temperatures, thereby, contributing to decreased sensitivity of plants to mesotrione. To test these hypotheses, we extracted total RNA from mesotrione-treated (105 g ai ha^-1^) plants grown at LT, OT, and HT, and quantified the *HPPD* expression levels using qPCR.

**Fig 6:**
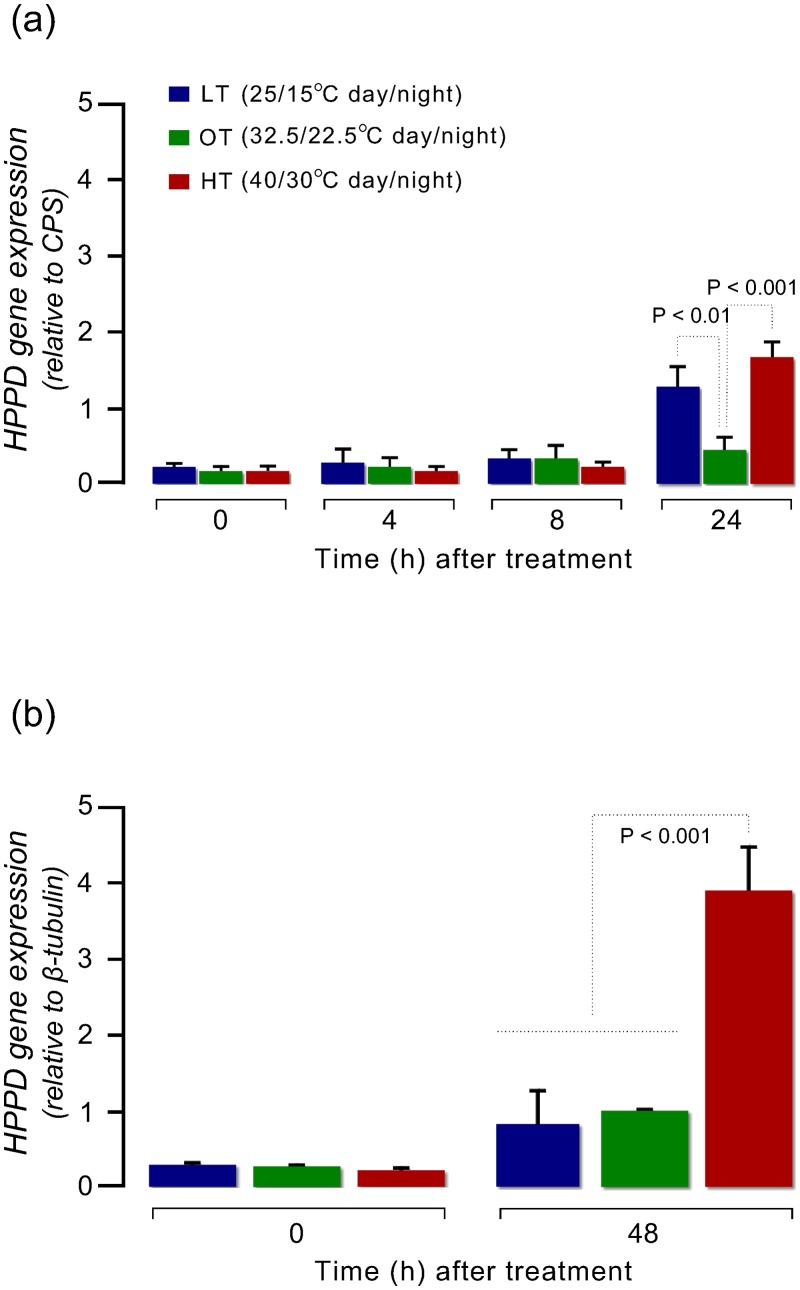
Time course of *HPPD* expression levels (a) relative to *CPS* and (b) *β*-tubulin in mesotrione-treated (105 g ai ha^-1^, 0.85% w/v AMS, and 1% v/v COC) Palmer amaranth leaves under three different temperatures (low temperature, LT, 25/15°C; optimum temperature, OT, 32.5/22.5°C; and high temperature, HT, 40/30°C; 15/9 h day/night). Total RNA was extracted from a bulked sample of Palmer amaranth leaves. Data represent means of two to three biological samples. Error bars represent SE.

Expression of *HPPD* relative to *CPS or β*-tubulin did not differ among untreated plants grown under different temperatures (P = 1) (Fig 6). Relative *HPPD*:*CPS* expression was similar among LT, OT and HT at 4 and 8 HAT (P > 0.99), and was not different from the level of expression in untreated plants (P > 0.9) (Fig 6a). Overall, the relative *HPPD* expression increased at 24 HAT compared to previous time points regardless of the growth temperatures (P < 0.01). At 24 HAT, the relative *HPPD* expression in plants grown under HT (1.63) was significantly higher than under OT (0.38) (P = 0) which is 15-fold higher compared to untreated plants (P = 0; 0.11 for untreated plants). At 48 HAT, relative *HPPD*:*β*-tubulin expression was 0.84 and 0.99 in LT and OT plants, respectively, whereas in plants grown under HT, the expression was 4-fold greater compared to LT and OT (P < 0.01), which is 18-fold higher compared to untreated plants (Fig 6b).

## Discussion

Our results showed a pronounced effect of growth temperature on mesotrione efficacy in Palmer amaranth. Consistent with previous findings in other weeds [26,34], Palmer amaranth’s sensitivity to mesotrione drastically increased when temperature decreased from 32.5/22.5 (OT) to 25/15°C day/night (LT) (Figs 1 and 2, S1 Fig; S1, S2 and S3 Tables). Documentation of the effect of elevated temperature (> 35°C) on efficacy of herbicides including mesotrione is lacking. In our study, Palmer amaranth was less sensitive to mesotrione when grown under HT (40/30°C day/night) compared to OT and LT.

Mesotrione absorption, translocation, and metabolism in Palmer amaranth were affected by growth temperatures. In tolerant crops, maize and sorghum (*Sorghum bicolor* L. Moench), rapid metabolism within the leaf limits translocation of mesotrione to other parts of the plant [43,44] which may explain greater absorption and metabolism of mesotrione at HT compared to LT and OT (Figs 3, 4 and 5). In addition, greater translocation of radioactive mesotrione, presumably comprising parent mesotrione, towards growing point at LT and OT compared to HT may contribute to increased sensitivity of Palmer amaranth at those temperatures. The translocated amount of mesotrione to the above-treated part was substantially lower compared to the amount absorbed. However, considering mesotrione’s potency to inhibit HPPD (*K*_*d*_ 15 *p*M) [41], a small amount of intact (non-metabolized) mesotrione may have a large effect on phytotoxicity.

It is well known that ROS-triggered signaling activates antioxidant systems and influence expression of many nuclear-encoded genes including those that encode for ROS scavengers and CYPs [45–50]. In this study, two stress factors, temperature (low and high) and mesotrione treatment, were imposed on Palmer amaranth plants. However, this study was limited to only the measurement of the expression of the target site gene of mesotrione. The expression of *HPPD* relative to *β*-tubulin was higher in the mesotrione-treated plants at HT, particularly at 48 HAT, which possibly contribute, to some extent, to minimize mesotrione toxicity; directly by reducing ROS production and/or indirectly by helping to quench excess ROS. On the contrary, the *HPPD* expression was similar at LT and OT, suggesting no role of the target site gene expression in the altered sensitivity to mesotrione. Although, the expression of *HPPD* was not influenced by the temperatures alone (within the range of temperatures used in this study), it is expected that many other temperature stress-response genes may have differentially expressed under different growth temperatures. Thus, apart from the rapid detoxification of the mesotrione and up-regulation of *HPPD*, high temperature-induced *in vivo* levels of antioxidant enzymes [49,51] may also have contributed to detoxify mesotrione-induced ROS in Palmer amaranth. Such cross-adaptation to subsequent or simultaneous stressors have been shown in plant species [52–54]. However, these results raise the question of how plants would respond when they were exposed simultaneously or in a shorter interval to high temperature and mesotrione.

In field, a wide array of other abiotic conditions, which vary in duration and extent, may exist leading to multiple stress conditions. During summer crop growing seasons, the high temperature conditions are often associated drought stress [55]. It would be of great interest to know whether the combined effect of drought and high temperature stress would provoke even stronger cross-adaptation to mesotrione application. Future studies should focus on deeper understanding of the outcome of interactions of multiple stresses on control of key agricultural weeds with mesotrione and other herbicides. Such information will open new possibilities for a predictable approach for effective weed management. Effective weed control is also crucial for sustained utility of currently available herbicides as reduced efficacy of herbicides facilitate resistance evolution [56–61].

## Supporting Information

S1 Fig Whole-plant mesotrione dose-response of Palmer amaranth mortality under low (LT, 25/15°C day/night), optimum (OT, 32.5/22.5°C day/night) and high (HT, 40/30°C day/night) temperature (15/9 h day/night) 4 weeks after treatment. (DOCX)

S1 Table Mesotrione dose-response analysis of Palmer amaranth height under low (LT, 25/15°C day/night), optimum (OT, 32.5/22.5°C day/night) and high (HT, 40/30°C day/night) temperature (15/9 h day/night) 3 weeks after treatment. (DOCX)

S2 Table Mesotrione dose-response analysis of Palmer amaranth visual injury under low (LT, 25/15°C day/night), optimum (OT, 32.5/22.5°C day/night) and high (HT, 40/30°C day/night) temperature (15/9 h day/night) 3 weeks after treatment. (DOCX)

S3 Table Mesotrione dose-response analysis of Palmer amaranth survival under low (LT, 25/15°C day/night), optimum (OT, 32.5/22.5°C day/night) and high (HT, 40/30°C day/night) temperature (15/9 h day/night) 4 weeks after treatment. (DOCX)

S4 Table Mesotrione dose-response analysis of chlorophyll index in Palmer amaranth leaves under low (LT, 25/15°C day/night), optimum (OT, 32.5/22.5°C day/night) and high (HT, 40/30°C day/night) temperature (15/9 h day/night) 2 weeks after treatment. (DOCX)

S5 Table Mesotrione dose-response analysis of PSII efficiency (*F_v_/F_m_*) in Palmer amaranth leaves under low (LT, 25/15°C day/night), optimum (OT, 32.5/22.5°C day/night) and high (HT, 40/30°C day/night) temperature (15/9 h day/night) 2 weeks after treatment. (DOCX)

S6 Table Analysis of rate of [^14^C] mesotrione metabolism in Palmer amaranth plants under low (LT, 25/15°C day/night), optimum (OT, 32.5/22.5°C day/night) and high (HT, 40/30°C day/night) temperature (15/9 h day/night) as determined by reverse-phase HPLC. (DOCX)
