# Supplementary material for: Physiological and Molecular Mechanisms of Differential Sensitivity of Palmer Amaranth (Amaranthus palmeri) to Mesotrione at Varying Growth Temperatures
Source: PLoS One. 2015 May 19;10(5):e0126731. doi: 10.1371/journal.pone.0126731 (PMC4437998; doi:10.1371/journal.pone.0126731)
Supplement: S4 Table — (DOCX) [file pone.0126731.s005.docx]

**S4 Table.** **Mesotrione dose-response analysis of chlorophyll index in Palmer amaranth leaves under low (LT, 25/15ºC day/night), optimum (OT, 32.5/22.5ºC day/night) and high (HT, 40/30ºC day/night) temperature (15/9 h day/night) 2 weeks after treatment.** Palmer amaranth plants (8-10 cm tall, 8-leaf stage) were treated with 0, 3.28, 6.563, 13.125, 26.25, 52.5, 105, and 210 g ai ha^-1^ mesotrione with 1% v/v crop oil concentrate (COC) and 0.85% w/v ammonium sulphate (AMS). Chlorophyll index was measured in fourth-fully expanded leaf from top of the plant using a chlorophyll meter (SPAD-502 Plus). ED_50_ and ED_85_ values for each growth temperature were estimated by a three parameter Weibull regression analysis as described by Knezevic et al. (2007). Values in parenthesis are SE and asterisks represent P < 0.001.

| Temperature | ED_50_ | ED_85_ | SI (ED_50_) | SI (ED_85_) |
| --- | --- | --- | --- | --- |
|  | -------- g ai ha^-1^ -------- | |  |  |
| LT | 6.30 (0.5) | 11.5 (1.2) | 4.22* | 5.28* |
| OT | 26.6 (2.3) | 61.1 (10) | 1 | 1 |
| HT | 73.4 (5.6) | 145 (15) | 0.36* | 0.42* |

ED_50_ and ED_85_ are the mesotrione rates that caused 50% and 85% reduction in chlorophyll index, respectively. SI is the sensitivity index based on the ratio of ED_x_ for OT and ED_x_ for LT or HT, where x is the ED_50_ or ED_85_ value.
