# Supplementary material for: Physiological and Molecular Mechanisms of Differential Sensitivity of Palmer Amaranth (Amaranthus palmeri) to Mesotrione at Varying Growth Temperatures
Source: PLoS One. 2015 May 19;10(5):e0126731. doi: 10.1371/journal.pone.0126731 (PMC4437998; doi:10.1371/journal.pone.0126731)
Supplement: S6 Table — (DOCX) [file pone.0126731.s007.docx]

**S6 Table.** **Analysis of rate of [^14^C] mesotrione metabolism in Palmer amaranth plants under low (LT, 25/15ºC day/night), optimum (OT, 32.5/22.5ºC day/night) and high (HT, 40/30ºC day/night) temperature (15/9 h day/night) as determined by reverse-phase HPLC.** All Palmer amaranth plants (10-12 cm tall, 8-leaf stage) were treated with 8- x 2.5-µL droplets (1.6548 mM mesotrione, 0.85% w/v AMS, and 1% COC) containing 7.2 kBq of [^14^C] mesotrione on the upper surface of fourth and fifth youngest leaves. T_50_ and T_80_ values for each growth temperature were estimated by a three parameter log-logistic regression analysis as described by Knezevic et al. (2007). Values in parenthesis are SE and asterisks represent *, P < 0.05; **P < 0.001.

| Temperature | T_50_ | T_80_ | SI (T_50_) | SI (T_80_) |
| --- | --- | --- | --- | --- |
|  | -------- g ai ha^-1^ -------- | |  |  |
| LT | 21.1 (2.68) | 53.9 (2.53) | 0.78 | 0.64** |
| OT | 16.5 (4.00) | 34.4 (5.34) | 1 | 1 |
| HT | 13.0 (1.30) | 23.9 (3.29) | 1.27 | 1.44* |

T_50_ and T_80_ are the times (h) after treatment required to metabolize 50% and 80% of the parent compound, respectively. SI is the sensitivity index based on the ratio of T_x_ for OT and T_x_ for LT or HT, where x is the T_50_ or T_80_ value.
